# Study of rip currents as one of the causes of swimmers' drowning in the Caspian Sea 

**Published:** 2019-07

**Authors:** Sareh Ghasemi Nejad, Ali Nazmi

**Affiliations:** ^*a*^Guilan Education Office - Sangar Area, Guilan, Iran.; ^*b*^Guilan Agricultural Research and Education Center, Guilan, Iran.

## Abstract

**Background:**

Coastal currents are among the main causes of death of professional swimmers. Sea waves moving to the beach always move a large amount of water to the land. The large amount of water returns to the sea. For this purpose, some waterways are developed seaward, where the water flows at high speed, opposite the waves’ direction (from the beach to the sea). These currents are completely different from the water waves, and they are called coastal currents, rip currents, or deadly currents, causing many casualties in many parts of the world, especially in the Caspian Sea.

**Methods:**

The present paper examines the rip currents and the methods for identifying these currents, how to deal with them and prevent drowning in the sea.

**Results:**

The results of the study show the area in which the wave is broken is not suitable for swimming, and the sea is usually calmer outside the waves’ breaking areas (seawards).

**Conclusions:**

A swimmer who can keep on the surface of the water would be safe, but when the swimmer is on the way of the current, he/she must never counteract it and swim in the opposite direction. The swimmer should float along the rip current and enter the outside of the wave breaking area seawards.

**Keywords:**

Rip currents, Swimming, Caspian Sea

